# Current Status of Early Blight Resistance in Tomato: An Update

**DOI:** 10.3390/ijms18102019

**Published:** 2017-09-21

**Authors:** Pragya Adhikari, Yeonyee Oh, Dilip R. Panthee

**Affiliations:** 1 Department of Horticultural Science, North Carolina State University, Mountain Horticultural Crops Research and Extension Center, 455 Research Dr., Mills River, NC 28759, USA; padhika2@ncsu.edu; 2 Center for Integrated Fungal Research, Department of Plant Pathology, North Carolina State University, Raleigh, NC 27606, USA; yoh2@ncsu.edu

**Keywords:** *Alternaria solani*, early blight, polygenic inheritance, quantitative trait loci

## Abstract

Early blight (EB) is one of the dreadful diseases of tomato caused by several species of *Alternaria* including *Alternaria linariae* (which includes *A. solani* and *A. tomatophila*), as well as *A. alternata.* In some instances, annual economic yield losses due to EB have been estimated at 79%. *Alternaria* are known only to reproduce asexually, but a highly-virulent isolate has the potential to overcome existing resistance genes. Currently, cultural practices and fungicide applications are employed for the management of EB due to the lack of strong resistant cultivars. Resistance sources have been identified in wild species of tomato; some breeding lines and cultivars with moderate resistance have been developed through conventional breeding methods. Polygenic inheritance of EB resistance, insufficient resistance in cultivated species and the association of EB resistance with undesirable horticultural traits have thwarted the effective breeding of EB resistance in tomato. Several quantitative trait loci (QTL) conferring EB resistance have been detected in the populations derived from different wild species including *Solanum habrochaites*, *Solanum arcanum* and *S. pimpinellifolium*, but none of them could be used in EB resistance breeding due to low individual QTL effects. Pyramiding of those QTLs would provide strong resistance. More research is needed to identify additional sources of useful resistance, to incorporate resistant QTLs into breeding lines through marker-assisted selection (MAS) and to develop resistant cultivars with desirable horticultural traits including high yielding potential and early maturity. This paper will review the current understanding of causal agents of EB of tomato, resistance genetics and breeding, problems associated with breeding and future prospects.

## 1. Introduction

Tomato (*Solanum lycopersicum* Linnaeus), native to the Andean region of South America, is one of the most common horticultural crops and cultivated throughout the world. It can be grown in a wide range of climates from tropical to temperate; it also can be cultivated under cover conditions when outdoor temperatures are not favorable. Tomato is the world’s second most consumed vegetable after potato [1]. The total world production of tomato is 161.7 million metric tons with a value of ∼$59 billion. USA tomato production contributes 13.2 million metric tons with a value of $5 billion to the total world production [2]. The USA ranks in third position in the total world production of tomato after China and India [2]. Tomatoes are consumed in several ways: fresh, mixed in other food items or processed and canned as sauce, ketchup, juice, salsa, paste, soup and pickled. Tomato is the richest source of vitamin A and C and supplies a sufficient amount of the antioxidant lycopene pigment that helps to protect the body against cancer and heart disease [3,4]. Because of its wide use and nutritional values, there is a high demand for both fresh market and processed tomato varieties. Higher production of tomato is therefore required to fulfill the ever-increasing demand.

However, the commercial production of tomato has been hindered by many fungal, bacterial, viral and nematode diseases including early blight. The cultivated tomato has a narrow genetic diversity that resulted from its intense selection and inbreeding during evolution and domestication [5]; thus, these species are more prone to disease epidemics. On the other hand, wild species are comparatively more resistant to diseases than cultivated tomato species. Therefore, the hindrance caused by several diseases can be tackled through the development of resistant cultivars by plant breeding approaches utilizing resistance in the wild species. This paper discusses the early blight (EB) disease and the pathogen, genetic studies and breeding of tomato, problems associated with breeding, QTL (Quantitative Trait Loci) mapping efforts for early blight resistance and prospects to improve breeding for EB resistance.

## 2. Early Blight 

### 2.1. Causal Agent

The genus *Alternaria* is large and consists of several economically-important plant pathogens including *A. solani*, *A. alternata* and *A. brassicicola*. Many species of *Alternaria* are significant causes of necrotrophic diseases of crops. For most species including *A. solani*, no sexual stages have been reported. *Alternaria* produce unique club-shaped conidia, often beaked with horizontal and often vertical septa that may be produced either individually or in a chain, depending on the species. Hyphal cells are darkly pigmented with melanin, which guards hyphae and spores against environmental stress and allows spores to survive in soil for long periods of time [6].

The taxonomy of *Alternaria*, particularly as it relates to EB, is undergoing revision. In 2000, Simmons recognized new species among the *A. solani*-like isolates from Solanaceae hosts. Based on cultural and morphological differences, Simmons proposed *Alternaria tomatophila* as “the common and widely distributed causal agent of early blight of tomato” [7]. More recently, Woudenberg et al. [8] grouped several large-spored isolates including those from Solanaceae, Cucurbitaceae and Scrophulariaceae into a new species designated *A. lineariae* [8]. Large spore species were distinguished from small spore forms including *A. alternata* and *A. arborescens* (*A. alternata* fsp *lycopersici*), which have also been isolated from solanaceous plants and have been reported to cause EB-like symptoms in some situations [9]. To differentiate these closely-related large-spored *Alternaria* species, several morphological, molecular and chemotaxonomic approaches have been applied. 

*A. solani* and *A. tomatophila* are morphologically very similar. Measuring the size of conidia and beak length in various media showed that *A. tomatophila* was generally shorter and had a thinner body with a longer beak than *A. solani*. However, due to variation within the same species, it was not possible to draw definitive conclusions [10]. Based on a detached leaf assay on both potato and tomato, *A. solani* isolates showed similar aggressiveness in both tomato and potato, while *A. tomatophila* was highly aggressive in tomato, but weaker in potato [10].

Molecular techniques have been developed for detecting and distinguishing *A. solani* from complex environments. Using the internal transcribed spacer (ITS) region, a PCR method was developed for the detection of *Alternaria* species from raw and processed tomato samples [11]. Furthermore, the primer sets designed from the coding region of the β-tubulin gene (*AS1* and *AS2*) and cytochrome *b* were successfully used for the specific detection of *A. solani* from the closely-related *Alternaria* species including *A. tomatophila* in tomato and potato [12,13].

Several studies support host speciation in *A. solani*. Amplified fragment length polymorphism (AFLP) analysis conducted on 112 *A. solani* isolates from Cuba and other countries around the world [14] showed that there was no significant correlation between genetic markers and the geographic origin of isolates. However, the analysis showed that isolates were separated mainly according to the host plant of origin. One major cluster contained 62% of isolates from potato, whereas the other major cluster contained 87% from tomato.

Similarly Gannibal, Orina, Mironenko and Levitin [10] analyzed sixty three large-spored *Alternaria* isolates from different hosts, mainly potato and tomato and a few from wild potato species in Russia, and compared them with representatives of *A. solani* and *A. tomatophila* collected in the USA [15]. Sequence analysis of *Alternaria* major allergen (*Alt a1*), glyceraldehyde 3-phosphate dehydrogenase (*gpd*) and calmodulin revealed six, four and four single-nucleotide polymorphism (SNP) markers, respectively. Haplotype analysis showed two major groups representing *A. solani* and *A. tomatophila* [10]. Furthermore, the majority of *A. solani* from the potato cluster had mating type locus 1 (*MAT1*) idiomorph *MAT1-1*, while 88% of *A. tomatophila* isolates had the *MAT1-2* idiomorph.

Secondary metabolite profiling has been used to distinguish large-spored *Alternaria* species, *A. dauci*, *A. porri*, *A. solani* and *A. tomatophila* [15]. Metabolite cluster analysis of 56 isolates confirmed *A. dauci*, *A. solani* and *A. tomatophila* as three distinctive species with their own metabolite profiles. Furthermore, this study revealed that altersolanol A, altertoxin I and macrosporin are produced both in *A. solani* and *A. tomatophila*, but not in *A. dauci*. Another chemotaxonomic approach of proteins, using matrix-assisted laser desorption ionization time of flight mass spectrometry (MALDI-TOF MS) of 37 isolates showed three major clusters corresponding mainly to *A. dauci*, *A. porri* and *A. solani*/*A. tomatophila*, but was able to distinguish *A. solani* from *A. tomatophila* [16].

Recently, the genome sequence of *A solani*, *A. tomatophila* and several other *Alternaria* species has been made publicly available [17]. Future comparative genome analyses will likely shed additional light on the molecular basis for host specialization between these closely-related pathogens. In the following sections, we will primarily focus on *A. solani*, recognizing that several others closely-related *Alternaria* species can cause EB of tomato.

### 2.2. Disease Cycle

**Figure 1:**
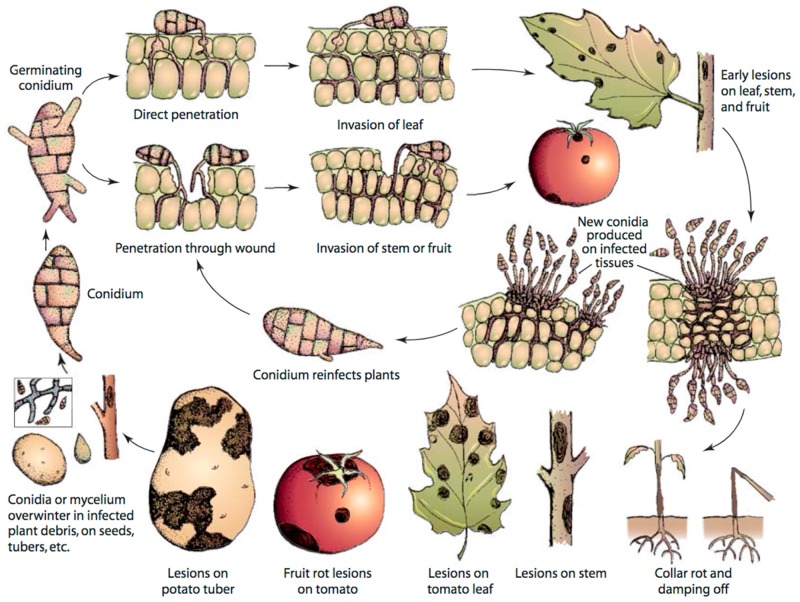
Disease cycle of early blight caused by *Alternaria solani* in tomato, adapted from Agrios [22].

*A. solani* reproduces asexually; a sexual stage of this fungus is unknown. The fungus overwinters in soil, plant debris, seed and alternate hosts in the form of either conidia or mycelia, which may serve as primary sources of inoculum (Figure 1). The thick cell wall of conidia enables the fungus to adapt to adverse climatic conditions [18]. Infection occurs during warm and humid conditions. Conidia germinates at temperature of 8–32 °C in cool and humid conditions in the presence of moisture to form germ tubes [19,20]. Germ tubes penetrate host tissue directly or enter through stomata or wounds, thereby causing infection. Lesions appear after 2–3 days of infection depending on environmental conditions, leaf age and cultivar susceptibility, and spores are produced 3–5 days after the appearance of lesions [19,20,21]. Generally, a long period of wetness is needed for spore production, but spores are also produced during alternate wet and dry conditions. First, conidiophores are developed during wet nights, which then produce spores or conidia in another wet night after the period of day light and dryness. In the next step, conidia are rapidly dispersed through wind and rain splash and continue the disease cycle in other healthy parts of the same plant or different plants. EB has the potential of causing polycyclic infection because of its short disease cycle [19,20].

### 2.3. Variability among Isolates

Despite having only asexual reproduction, isolates of *A. solani* highly differ in their morphology, toxin production and genetic composition. Such variation might have arisen from heterokaryosis and natural mutation [23]. Morphological variations among *A. solani* isolates are observed in terms of types of conidia (beaked or non-beaked), length of conidiophores, dimension of the spore body and vegetative compatibility. Morphological variations are often affected by environmental factors such as substrate, light intensity and temperature. Therefore, other variations should be taken into account while differentiating among species in addition to morphological variation. High variations among isolates have been detected at the molecular level using several molecular markers. In addition, *A. solani* also possesses extensive variation in terms of its toxin production. Several *Alternaria* species, which were difficult to distinguish by their morphological characteristics, have been classified based on their toxin production. Nonetheless, differentiation of *A. solani* based on toxin production is still not unanimous among researchers and scientists [18].

Because of high genetic variation, *A. solani* can easily adapt to the changing environment and develop resistance to fungicides [24]. Furthermore, high genetic variation also has a higher risk of overcoming existing genetic resistance of the host, thus challenging the control of EB and the development of a completely EB-resistant cultivar [25]. However, there are no physiological races of *A. solani* known or confirmed so far, suggesting host non-specific recognition of the pathogen. This makes the studies of the pathology and genetics of EB more complex [18]. 

### 2.4. Molecular Mechanism of Infection

Fungal pathogens produce a number of enzymes and secondary metabolites that help to infect the host plant. The secondary metabolites may kill or activate the host cells and penetrate into the cells before invasion. *A. alternata* has been reported to produce AAL toxin and causes *Alternaria* stem canker in tomato [26]. A polyketide synthase gene (*PKS*) *ALT1* was involved in producing the AAL toxin biosynthesis. Additional toxins associated with *Alternaria* sp. infection have been reported such as Brefeldin, tentoxin, ACT toxin, AF-toxin, AK-toxin, ACTG-toxin, ACR-toxin, AM-toxin, AS-toxin and maculosin destruxin B [27]. Enzymes involved in the pathogen establishment on the host tissue surface include cutinases, lipase, endo-glucanases and exo-glucanases [27].

To date, limited studies have been conducted to determine the molecular basis of infection and host responses to *A. solani*. Many plant pathogenic fungi including *Alternaria* species produce phytotoxins. Based on selectivity, phytotoxins are classified as either host-specific toxins (HSTs) or non-host-specific toxins (NST). *Alternaria* species produce a variety of HSTs, and most are low molecular weight secondary metabolites. The HTSs are structurally diverse and some are involved in pathogenicity or virulence during host-plant interaction, as has been shown in *A. alternata* [27,28]. 

*A. solani* produces various types of phytotoxic metabolites, including alternariol, altersolanol A, altertoxin, macrosporin and solanapyrone [15,29]. Secondary metabolite profiling of large-spored *Alternaria* species revealed that *A. solani*, as well as *A. dauci*, *A. porri* and *A. tomatophila* strains produce common secondary metabolites including altersolanol A, altertoxin I and macrosporin, as well as unknown *A. solani*-specific secondary metabolites [15]. The role they play in disease has generally not been determined, with a few exceptions.

Several forms of solanapyrone A, B and C are produced in *A. solani*. Solanapyrone is derived from a biosynthetic gene cluster consisting of six *sol* genes encoding a polyketide synthase, an *O*-methyltransferase, a dehydrogenase, a transcription factor, a flavin-dependent oxidase and cytochrome P450 [29]. *Sol5* encodes Diels-Alderase, which is involved in the last step of solanapyrone biosynthesis. *Sol5* gene knock out mutants were unable to synthesize solanapyrone, but showed no change in virulence compared to the wild type. Thus, despite the strong phytotoxicity of solanapyrone, it is not required for pathogenicity by the fungus [30].

Extracellular proteases are key enzymes produced by fungi during the host-pathogen interaction and are involved in growth, development, survival and pathogenicity [31]. The presence of an extracellular serine protease and metalloprotease was recently found to be secreted by *A. solani* and proposed to be involved in phytopathogenicity [32,33]. However, additional molecular studies are needed to demonstrate that they play a role in virulence.

Chitin is the major component of most fungal cell walls. Plants express chitinases during fungal infection and are recognized as pathogenicity-related (PR) proteins as part of the defense response against fungal attack [34]. Against *A. solani*, plant chitinases produce strong antifungal effects, and transgenic expression of an endo-chitinase in potato provided disease resistance [35]. Similarly, transgenic expression of a rice chitinase gene in tomato enhanced disease resistance to early blight [34].

Microarray expression profiling has been used to investigate differential gene expression in susceptible and resistance tomato varieties in response to *A. solani* [36]. A number of genes including members of the ethylene responsive transcription factor (ERF) family, ribosomal protein transcription factors and zinc finger transcription factors were responsive in resistance tomato. Such genes have been proposed to play a role in plant defense against *A. solani* possibly by activating PR protein synthesis [36]. However, direct evidence for such a role remains to be determined.

*A. solani* is sensitive to quinone outside inhibitor, strobilurin and fungicides. However, resistance is being encountered. An amino acid substitution at position 129 in *A. solani* cytochrome *b* (so-called F129L, where phenylalanine is changed to leucine) is responsible for the decreased sensitivity [37]. A single-nucleotide polymorphism responsible for sensitivity has been shown to be increasing rapidly in populations in recent years [38].

Recently, a whole genome sequence for *A. solani*, as well as several other key *Alternaria* species has been published [17]. Based on Illumina whole genome sequencing technology, *A. solani* (BMP 0185) was predicted to contain 11,726 genes in a 32.9-Mb genome. In the coming years, comparative and functional genome analysis among *Alternaria* spp. will help shed new light on the molecular mechanism of disease caused by *A. solani*.

### 2.5. Disease Symptoms

The symptoms of *A. solani* appear in leaf (leaf blight or early blight), stem (collar rot or stem lesions) and fruit (fruit rot) of tomato plant [21,39]. Leaf blight is the most important phase of the disease. It is characterized by dark, small, necrotic, coalescing and concentric lesions giving a target-like appearance on the leaf surface. The lesions are surrounded by yellow rings [21]. The disease first appears on lower older leaves and moves upward as the plant becomes mature [6]. Older leaves are more susceptible than younger leaves. As disease progresses, heavy defoliation occurs, raising the respiration rate and lowering the photosynthetic rate. Defoliation also exposes fruits to the Sun, causing sunscald. This leads to poor fruit quality and significant yield loss [18].

Collar rot is typified by initial dark and sunken lesions on the stem, which later expand to form lens-shaped lesions with concentric rings similar to those on leaves. Lesions at the ground level girdle the stem in young seedlings, destroy the vascular system and form “collars” [18]. Collar rot also serves as a source of inoculum.

The infection on fruit causes dark, sunken, leathery and purple lesions on the stem-end. These lesions expand to a significant size and extend deep into the flesh of the fruit. Infected fruits mostly drop prematurely, and those reaching to maturity also become unmarketable [23].

### 2.6. Control Measures

EB can be controlled by three measures: cultural practices, fungicide treatment and the use of resistant varieties. Cultural practices and fungicidal treatment are more common practices [18]. Cultural practices include maintenance of a healthy field and crop vigor, sanitation, removing infected vines and fruits, plant debris and volunteer weeds from the vicinity of the field, crop rotation and reducing the leaf wetness by soil-directed irrigation systems [18,23]. However, cultural practice alone is not sufficient for the control of EB.

Several types of fungicides have been developed for the control of EB, but fungicide treatment is not economically feasible, nor environmentally sound. Fungicides are first applied 1–2 days after transplantation and then require routine application at the interval of 7–10 days for effective control, thereby increasing production cost and environment pollution [18,20]. In North Carolina, a total of 15 applications of fungicides is suggested per growing season including pre-harvest and during the harvest period [40]. Fungicides often fail to work under high disease pressure [18]. The frequent use of fungicide also results in the emergence of new fungicide-resistant isolates due to high selection pressure.

The use of biological controls has also been documented in several research papers as an alternative method for the management of EB. The four species of *Pseudomonas*—*P*. *fluorescens*, *P. aeruginosa*, *P. putida* and *P. cepacia*—have resulted in less disease severity and higher fruit weights compared to control in tomato [41]. Similarly, *P. gladioli* B25 reduced EB disease by 60.2% by inducing systemic resistance in the host due to an enhanced level of phenol accumulation, peroxidase activity and PALase activity [42]. Extracts of several plants such as *Cinnamomum zeylanicum*, *Syzygium aromaticum*, *Ferula foetida*, *Inula racemosa*, *Hemidesmus indicus*, *Rubia cordifolia*, *Glycyrrhiza glabra* and *Saussurea lappa* also showed antifungal activity against EB pathogen ranging from a medium to a high level [43]. In addition, the role of fungi such as *Trichoderma viride* [44] and *T. harzianum* [45] in the management of EB has been reported. The plant growth-promoting rhizobacteria *Bacillus subtilis* also increased systemic resistance in tomato by inducing growth hormones and defense-related enzymes such as peroxidase, polyphenol oxidase and superoxide dismutase [45]. Other antagonistic bacillus strains (3F-II, 3F-VII, 13F-III, 15F-III) decreased EB severity on tomato, which could be due to an antibiosis-like mechanism or ecological competition [46].

The association of the microbial community including mycorrhiza with plants’ growth and development has been known for a long time. These microbes produce different types of hormones including auxins and cytokinins and impact plant growth [47,48,49]. A new approach at the holobiont level is one in which microbial communities are also considered in the plant selection process [50]. This will ultimately help to improve the overall performance of plants under field conditions. While this is a relatively new approach, targeting the microbial community for the improvement of plant performance, it may be a novel approach and can be exploited for the overall performance of plants to address biotic and abiotic stresses in plants [51,52].

Use of resistant varieties is the most provident, effective and sustainable control measure of EB of tomato. The genetic integration of effective EB resistance into cultivated tomato would reduce dependence on chemical inputs and have significant environmental, health and financial benefits.

## 3. Screening Methods

Disease screening plays a key role in the identification of resistant lines from diverse germplasm, which then can be used as a source of resistance in breeding programs. Proper disease screening methods are required in disease resistance breeding to get reliable and accurate data. Disease symptoms can be evaluated in the field, greenhouse and growth chamber.

### 3.1. Inoculum Preparation

Inoculum of *A. solani* can be prepared in various culture media such as potato-dextrose agar (PDA) [53], V-8 juice agar [54], lima bean agar [55] and sporulation medium [56]. Sporulation is enhanced by mycelial wounding or transferring culture onto minimal media and incubating culture at 21–23 °C under a cool-white fluorescent diurnal light with a 12 h photoperiod [56,57]. The conidia are collected after 10–14 days by flooding the plates with ddH_2_O (containing 0.01% Tween 20) and brushing the agar surface with a paintbrush [58]. A spore concentration of 10,000–40,000 conidia/mL is maintained prior to inoculation using a hemocytometer. The inoculation methods may vary depending on the screening methods [59]

### 3.2. Field Screening

Field screening is the most common method of screening. It allows screening of a large number of populations at the same time under natural conditions and keeping track of disease severity in every phase of the plant life cycle [18]. EB severity in the field is usually evaluated by recording the final percent of defoliation and calculating the area under the disease progress curve (AUDPC) using the data observed over time [60]. AUDPC combines the effects of host, pathogen and environment [23] 

However, field screening has some disadvantages. It requires a large amount of inoculum, disease spreader rows and intensive labor for the evaluation of large populations. It is also difficult to maintain treatment uniformity in the field. The high disease pressure and confounding effects of other foliar diseases reduce the effectiveness of field screening [18]. To avoid such confounding effects, other foliar diseases should be eliminated using fungicides before the application of inoculum [61]. Besides, the environment may not be favorable for the disease incidence in every season in every location, and even if every condition is optimal, field screening allows only one cycle of screening per year, which decelerates the pace of breeding for EB resistance [18].

### 3.3. Greenhouse Screening 

Greenhouse screening is useful in regions where field screening is difficult due to an unfavorable environment and confounding effects of other complex diseases. As the environment can be controlled in the greenhouse, it provides a conducive, uniform and repeatable environment. Unlike field evaluation, greenhouse screening allows several cycles of screening in a year [18]. During greenhouse experiments, inoculated seedlings are first kept under high relative humidity (RH) (100% RH) for 24 h. This is then reduced to 14–16 h only at night for 5–7 days in a mist chamber to allow disease development. The severity of the disease is measured in terms of the percentage of necrotic area on leaves or the number of lesions when there are few necrotic spots usually after seven days of inoculation [57].

Several studies have been conducted in greenhouses with different results. Greenhouse studies involving moderately-resistant and susceptible lines did not show a significant difference in their symptoms [57,62], whereas those involving highly-resistant and susceptible lines showed significant differences in their symptoms [63]. This indicates that greenhouse screening may only be able to distinguish two extreme groups of genotypes. However, in the study conducted by Foolad, Ntahimpera, Christ and Lin [58], the greenhouse results were comparable with those of the field test, suggesting its use for initial screening to expedite the selection process. Greenhouse screening is not very common for genetic analysis studies [25].

Greenhouse screening is affected by several factors including plant age, inoculum quality and quantity, inoculation technique and pre- and post-inoculation environmental conditions [18]. As younger leaves are more resistant to EB than older and mature leaves, it is difficult to distinguish the true EB-resistant genotype seedling stage. Therefore, the greenhouse may be more suitable for the selection of collar rot-resistant lines [64]. However, plants at their late seedling and early flowering stage (7–8 weeks) can be successfully evaluated in the greenhouse [58]. In a study by Vloutoglou and Kalogerakis [65], with the increase in inoculum quantity from 6.2 to 11.5 conidia/mL, the defoliation percentage increased linearly. Likewise, the disease was developed after 4 h of leaf wetness, and the severity increased with the increase in the leaf wetness period up to 24 h, but did not increase further after 24 h [65].

### 3.4. Laboratory Assays (Detached Leaflet Assay)

The detached leaflet assay is performed in a growth chamber. Unlike field and greenhouse evaluation, it can only measure foliar resistance. A droplet of spore suspension is deposited on the upper surface of either punctured [53] or non-punctured [58], young, fully-expanded leaflets obtained from the greenhouse. After inoculation, leaflets are incubated in the dark for 24 h at 22 °C, followed by incubation under cool-white fluorescent diurnal light with a 12-h photoperiod [58]. In the study of Foolad, Ntahimpera, Christ and Lin [58], the detached leaflet assays did not correlate with either field or greenhouse results, but field and greenhouse results had a good correlation, suggesting the requirement of whole plant for the evaluation of EB resistance. On the other hand, Locke [53] obtained successful results with this method. In general, this method alone is not reliable for EB resistance evaluation, but can be used for comparing the virulence of different strains of *A. solani* and the evaluation of EB resistance along with other methods.

## 4. Genetic Source of Resistance

Cultivated tomatoes (*S. lycopersicum*) are mostly susceptible to early blight, and only a few resistant accessions are available. *S. lycopersicum* accession PI138630 has been used as a source of resistance in early EB-resistant breeding [57]. Two early blight breeding lines 71B2 (resistant to leaf blight, but susceptible to collar rot) and C1943 (highly resistant to collar rot and moderate resistance to leaf blight) were used for developing many EB-resistant tomato breeding lines: NC63EB, NC870, NCEBR-2, NCEBR-3, NCEBR-4, NCEBR-5 and NCEBR-6; as well as hybrids: “Plum Dandy”, “Mountain Magic”, “Mountain Merit” and “Mountain Supreme” [66,67,68,69,70]. Other varieties such as “Devon Surprise”, “Red Cherry”, “Red Pear 414”, “Red Pear 415”, “Targinnie Red”, “Vetomold”, “Montgomery”, “Norduke” and “Marglobe” have resistance to collar rot [71]. However, there are several identified highly and moderately EB-resistant accessions of *S. lycopersicum* that are not known to be used for EB resistance breeding in tomato such as *S. lycopersicum* f. sp. *cerasiforme* Accession PI406758 (highly EB resistant) [18].

Resistant sources have been identified in wild species *S. habrochaites*, *S. pimpinellifolium* and *S. peruvianum*, and utilized to develop resistant lines in a tomato breeding program [18]. *S. habrochaites* is the richest source of resistant genes among wild species of tomato, but these species are late maturing, indeterminate and low yielding. Some highly-resistant *S. habrochaites* accessions are: PI127827, PI126445, PI390513, PI390514, PI390516, PI390658, PI390660, PI390662, PI390663, LA2100 and LA2650 [72,73]. These resistant accessions have been used to develop other EB-resistant lines. *S. habrochaites* accession PI390662 was used to develop EB-resistant USDA Line 87B187 [57] and PI126445 to develop NCEBR-1 [66]. Similarly, B6013 was used to develop H-7, H-22 and H-25 EB-resistant lines [74]. The F_1_ hybrids of resistant accessions of *S. habrochaites* and the cultivated tomato showed a moderate to high degree of resistance, yet they also possessed other undesirable characteristics of *S. habrochaites* such as late maturity and small fruit size.

*S. pimpinellifolium* is a close relative of cultivated tomato, hence, it can be easily crossed with cultivated tomato. It also possesses fewer undesirable traits compared to other wild species of tomato. Therefore, *S. pimpinellifolium* has been frequently used in tomato breeding programs. Several highly- and moderately-resistant *S. pimpinellifolium* accessions have been obtained in a field, greenhouse and growth chamber evaluation [18,75]. *S. pimpinellifolium* Accession LA1921 and its BC4F4 progeny exhibited high resistance towards EB [74].

Although some resistant accessions PI129152, PI127829, PE33, LA1292, LA1365, LA1910, LA1983 and PI270435, have been identified in *S. peruvianum*, they have not been fully utilized in tomato breeding for EB resistance because of high genetic variation within each accession and the cross-incompatible nature of *S. peruvianum* with cultivated tomato [57,73] All the identified genetic sources of resistance for EB disease are summarized in Table 1.

### Characterization of EB Resistance

EB resistance is a complex trait that is quantitatively inherited and controlled by the additive or non-additive interaction of multiple genes and their interaction with the environment [39,61]. No studies have detected a single qualitative gene for EB resistance. Tomato lines such as C1943, 71B2, IHR1939 and IHR1816 exhibited a polygenic recessive inheritance pattern [39,76,77]. Other lines such as NCEBR-1 and 83602029 showed dominant genes involving additive, dominance and epistatic effects, whereas NCEBR-2, 87B187 and NC39E had polygenic partial dominance [23]. The study of EB resistance is mainly focused on the leaf blight phase. Only a few studies have been conducted on the collar rot and fruit rot phase, where the inheritance of collar rot and fruit rot was independent of leaf blight inheritance [75,78]. The few studies on collar rot and fruit rot might be due to the requirement of a longer time compared to leaf blight, as the disease first appears on leaves.

A low to moderate heritability (*h*^2^) of 0.26–0.38 [61], 0.65–0.75 [59,79] and 0.43–0.72 [80] was detected for EB resistance. Therefore, breeding for EB resistance totally based on phenotypic selection will take a longer time. The marker-assisted breeding methods may accelerate the transfer of resistant genes from wild species to cultivated species, but no such markers for the selection of EB resistance are currently available. Some studies have investigated the physiological and molecular defense response of resistant tomato lines against *A. solani* compared to susceptible lines. EB resistance was associated with enhanced expression of *TPK1b* (a defense-related gene), increased accumulation of H_2_O_2_, phenolic compounds and superoxide anion, a higher percentage of cross-linking and polyphenol activity, decreased DNA damage and less reduction in cell viability [81]. These data might be useful in understanding the basis of EB resistance, hence to develop resistant tomato lines against EB [81].

## 5. Breeding for EB Resistance

Tomato breeding for various desirable traits began more than 100 years ago in the United States [82]. Several breeding lines with EB resistance have been developed utilizing resistance from wild species [39,57,66,67,68,70,74,83] (Table 1). Improving EB resistance through traditional breeding is difficult and challenging mainly because of two reasons: (i) the polygenic nature of resistance and (ii) the association of resistance with several undesirable characteristics such as physiological maturity, fruit load and plant type. Generally, late-maturing, low fruit-yielding and indeterminate tomato lines are more EB resistant, which has impeded the development of high EB-resistant cultivars with desirable traits through phenotypic selection [62,84,85]. In a cross between *S. habrochaites* and *S. lycopersicum*, resistant lines were obtained, but with late maturity, indeterminate growth and low yield, which were inherited from *S. habrochaites* accessions [23]. However, some recombinant inbred lines (RILs) that were derived from the cross of *S. pimpinellifolium* accession LA2093 and *S. lycopersicum* tomato breeding line NCEBR-1 were reported with EB resistance and other desirable characteristics including high yield, earliness in maturity and small plant size [86]. Although EB resistance in those RILs was partially affected by growth habit, plant size, maturity and fruit yield, no known genetic relationship between EB resistance and other horticultural traits could be detected in this study.

Younger leaves are more resistant to EB than older leaves because of high sugar content and glycoalkaloids (solanine, chaconine and solanidine) [6,87]. Therefore, a late maturing crop may appear EB resistant without even having resistant genes because of the high sugar content and glycoalkaloids [23,88]. Such a correlation of negative horticultural traits and EB resistance has created difficulty in phenotypic selection during tomato breeding.

The development of molecular markers opens the possibility of solving the problems seen in traditional breeding of EB resistance. Molecular markers, such as restriction fragment length polymorphisms (RFLPs), amplified fragment length polymorphisms (AFLPs), simple sequence repeats (SSRs), cleaved amplified polymorphic sequences (CAPS), resistance gene analogues (RGAs), expressed sequenced tags (ESTs) and conserved orthologs sets (COS), have already been mapped onto all 12 tomato chromosomes and used for locating QTLs governing important agricultural traits [1] (Table 2). The emergence of next generation sequencing (NGS) and high throughput genotyping platforms has enhanced the discovery of a large number of single nucleotide polymorphisms (SNPs). As SNPs can detect minor variation among populations and can exploit intraspecific variation, they are now the “marker of choice” in tomato breeding [91]. Such markers would be very useful in the dissection of complex quantitative EB resistance into individual genes, selection of an EB-resistant genotype at the early seedling stage and understanding the genetic basis of correlation between EB resistance and negative horticultural traits [80]. Therefore, quantitative trait loci (QTL) mapping efforts are being conducted to identify markers linked to QTL controlling the EB resistance. The location of QTLs governing different horticultural traits and EB resistance can then be compared to determine if the correlation is due to a pleiotropic effect or linkage drag [92]. Unfortunately, no such markers have been developed yet for the selection of EB resistance because of the lack of QTL strong enough to be used in breeding programs. The QTLs associated with EB resistance that have been identified so far are summarized in Table 2.

The first QTL mapping effort for EB resistance was performed on backcross populations (BC_1_ and BC_1_S_1_) resulting from a cross between susceptible *S. lycopersicum* (NC84173) and highly-resistant *S. habrochaites* (PI126445) [90]. In this study, the self-incompatible, late-maturing, indeterminate and low-yielding plants were removed from the BC_1_ population before EB evaluations to avoid confounding effects of those factors on EB resistance. Nine significant QTLs on chromosomes 1, 2, 5, 9, 10, 11 and 12 and two minor QTLs on chromosomes 8 and 11 were detected in the BC_1_ population for EB resistance using the simple interval mapping method. The individual effects ranged from 8.4% (chromosome 5) to 21.9% (chromosome 1). Simple interval mapping analysis also detected thirteen significant QTLs in the BC_1_S_1_ population on chromosomes 1, 2, 3, 5, 8, 9, 10, 11 and 12 for EB resistance, with individual effects varying from 7.9% (chromosome 5) to 25.9% (chromosome 9). The six QTLs out of 11 in the BC_1_ population on chromosomes 1, 5, 9 (two QTLs), 10 and 11 and, again, the six QTLs out of 13 located on chromosomes 1, 5, 8 (two QTLs), 9 and 10 in the BC_1_S_1_ population were confirmed as independent QTLs using the composite interval mapping method [90].

Zhang, Lin, Nino-Liu and Foolad [93] conducted a mapping study on the same BC_1_ population using a selective genotyping approach to detect and confirm QTLs governing EB resistance in PI 126445. They identified seven QTLs on chromosomes 3, 4, 5, 6, 8, 10 and 11; four of which, on chromosome 5, 8, 10 and 11, were also detected in the previous study of Foolad, Zhang, Khan, Nino-Liu and Lin [90], suggesting the potential utility of these QTLs in molecular breeding of tomato for EB resistance. All of the QTLs were inherited from a resistant parent except on chromosome 3.

Another study identified six QTLs governing EB resistance on chromosomes 1, 2, 5, 6, 7 and 9 that explained 7–16% of total phenotypic variance, using different environmental conditions and disease scoring methods in F_2_ and F_3_. F_2_ and F_3_ populations were obtained from a cross between EB-resistant *S. arcanum* (LA2157) and susceptible *S. lycopersicum* (Solentos) [25]. QTLs on chromosomes 2 and 7 were inherited from a susceptible parent. Three EB QTLs on chromosomes 2, 5 and 9 were detected in both field and glasshouse tests and also coincided with stem lesions, whereas two QTLs on chromosomes 1 and 6 were detected only in a field test, and one QTL on chromosome 7 was detected only in a glasshouse test. Only QTLs on chromosomes 2 and 9 were effective in both environments. QTLs on chromosome 9 also contributed 35% of total phenotypic variance of stem lesions; hence, they could be useful in breeding. The detected QTLs were also similar to QTLs detected in Foolad, Zhang, Khan, Nino-Liu and Lin [90] and Zhang, Lin, Nino-Liu and Foolad [93].

QTL mapping for EB resistance has also been conducted in a population derived from *S. pimpinellifolium*. After extensive screening of 300 accessions of *S. pimpinellifolium*, Accession LA2093 (previously designated as PSLP125) was selected for QTL mapping. LA2093 has good EB resistance plus other desirable characteristics [18,80,86]. First, the F_2_, F_3_ and F_4_ populations derived from the cross of LA2093 and tomato breeding line NCEBR-1 were used for QTL mapping, which detected 10 QTLs conferring EB resistance on chromosomes 2, 3, 4, 5, 6, 7, 9 and 12 with an individual effect of 7.6–13.4% and a combined effect of 44% of total phenotypic variance [18]. Out of ten QTLs, three were from NCEBR-1. This result was further verified using the F_2_ population of the same cross for QTL mapping through a selective genotyping approach, where nine EB QTLs were detected on chromosomes 1, 2, 3, 4, 5, 6 and 11 [18]. Therefore, these QTLs can be further utilized in EB resistance breeding.

Again, five major QTLs for EB resistance were identified on chromosomes 2 (two QTLs), 5, 6 and 9, using recombinant inbred lines (RILs), i.e., the F_7_, F_8_, F_9_ and F_10_ generations of the same cross (LA2093 x NCEBR-1) [80]. QTLs on chromosome 2 and 6 were from LA2093, whereas QTLs on chromosomes 5 and 9 were from NCEBR-1. QTLs on chromosomes 5 and 6 were most stable across generations with the largest effects that explained 11–17% and 10–16% of the phenotypic variation, respectively. Therefore, these two QTLs are important in EB resistance breeding. The detected QTLs were also co-localized with other resistant genes and candidate ESTs, suggesting the possibility of the detection of candidate genes for EB resistance after more research and investigation [80]. 

Several breeding efforts are ongoing to develop early blight-resistant varieties, but no commercial early blight-resistant variety has been reported yet [94]. The lack of qualitative genes and markers and the association of undesirable traits have limited the development of a resistant cultivar. Researches are also directed towards the transgenic tomato to increase the EB resistance. Arshad et al. [95] reported enhanced early blight tolerance with high fruit and nutritional quality in tomato plants transformed with the *rolB* gene of *Agrobacterium rhizogenes.* Babu, Jogaiah, Ito, Nagaraj and Tran [94] reported that the plant growth-promoting rhizobacteria (PGPR) are involved in the suppression of *A. solani.* Tomato plants pre-treated with PGPR showed improved resistance against early blight, which was associated with increased synthesis of antioxidant peroxidase (*POX*) and polyphenol oxidase (*PPO*) enzymes. This suggests the potential utility of *POX* and *PPO* genes to develop transgenic tomato lines with improved resistance against early blight [94].

## 6. Conclusions and Future Prospects

To conclude, EB resistance is a complex trait, governed by multiple genes, and yet, no pathogen races have been reported. There is no single gene-for-gene model for EB resistance and no major resistance gene identified. Furthermore, EB resistance is affected by different factors including physiological maturity, plant type, growth habit and fruit load. More importantly, the existing EB resistance in cultivars is not enough, and still, growers depend on cultural practices and extensive use of fungicides for the control of EB [86]. Although EB resistance has been identified in wild species, only a moderate level of resistance has been obtained in breeding lines and cultivars, and often, they are associated with undesirable horticultural traits like low yield and late maturity. Some desirable epistatic interactions between the QTLs and other segregating genes from wild germplasm may be lost while transferring alleles from wild to cultivated species, leading to the change in QTL effects in cultivated species [96]. Because of this, it is difficult to obtain full resistance as that of the original wild sources.

Breeding programs require stable QTLs having large additive effects and independent of epistatic interactions to be used in MAS. In the above studies, QTLs for EB resistance have been detected in almost all tomato chromosomes in different interspecific crosses. Among them, QTLs on chromosome 5 and 6 were reported in every study and have also been verified in a study by Foolad, Merk and Ashrafi [18]. QTLs on chromosome 5 and 6 were also the most stable QTL in a study of Ashrafi and Foolad [80]. However, the detected QTLs had small effects, large marker intervals, i.e., >10 cM, that may contain multiple closely-linked genes for the single trait, and only a few of the detected QTLs have been validated. It is also difficult to determine whether the detected QTL controls other traits or not. Further study of these QTLs and fine mapping might lead to important outputs in EB resistance breeding programs. Fine mapping allows precise estimation of the effects and chromosomal positions of the detected QTLs, which can be conducted by establishing introgression lines (ILs), near isogenic lines (NILs) and sub-NILs containing the resistance portion from wild species so as to incorporate resistant QTLs into cultivated tomato. Such lines also help to break down the possible negative correlation of the resistance QTLs with other undesirable traits prior to introgression in elite cultivars and to identify the physiological basis of QTL effects, pleotropic effects and multiple genetic factors controlling the trait [96]. 

Additionally, narrow genetic diversity among cultivated tomatoes, limited polymorphic markers for intraspecific populations, extreme labor for markers like AFLP and RFLP and the unavailability of reliable PCR-based markers are major problems in molecular breeding of tomato. Most genetic maps were based on interspecific crosses of cultivated tomato and wild species because of the greater number of polymorphic markers, but most breeding populations are based on intraspecific crosses of cultivated tomato or with that of the closely-related species *S. pimpinellifolium*, where there are limited marker polymorphisms [91]. The polymorphic markers selected from the wide crosses were not polymorphic within cultivated species [97].

With the development of next generation sequencing technologies and the decreased cost of sequencing, highly polymorphic markers such as SNPs can now be incorporated into the tomato breeding program, which can detect the polymorphisms among closely-related individuals within *S. lycopersicum* or between *S. lycopersicum* and closely-related species. The work in [98] identified one SNP per 8500 bases with 101 candidate SNPs within cultivated germplasm for 44 genes. Labate and Baldo [99] identified several SNPs among more than 15 tomato lines by EST mining and resequencing. Hamilton, Sim, Stoffel, Van Deynze, Buell and Francis [97] identified 62,576 SNPs through NGS of the transcriptome for six tomato accessions: three fresh market types, two processing, one cherry and one *S. pimpinellifolium*. Identification of SNPs and their utilization to uncover genetic variation among elite cultivars provides new insights into tomato breeding for EB resistance. 

In general, breeding for EB resistance in tomato without compromising other desirable horticultural traits is a demanding task. More research and investigations are needed to identify additional resistant sources, to validate, utilize and introduce detected QTLs in cultivated tomato species for the release of EB-resistant breeding lines and cultivars with high yield and early to mid-season maturity. 

QTLs have been identified in *S. habrochaites*, *S. pimpinellifolium* and *S. arcanum*. QTLs on chromosome 5 and 6 were stably observed across generations and environments with the effect of 10–16% [80] and also reported in every study, whereas the QTL on chromosome 9 explained the largest percentage of phenotypic variance (25.6%) for EB resistance in the *S. habrochaites* PI 126445 accession [90,93]. The QTL on chromosome 9 of the *S. arcanum* LA2157 accession was also stable and showed a major effect on the stem lesion resistance, i.e., 35% of total phenotypic variance [25]. However, none of these QTLs have been fine mapped. Therefore, research can focus on these chromosomes with the largest QTL effects in the future. Fine mapping of these QTLs will allow the determination of the precise location of these QTLs and the prevention of the introgression of other undesirable regions of the donor genome. Since the detected QTLs have less individual effects, QTLs from different species can be combined after fine mapping and QTL validation in a single cultivar via gene pyramiding to provide strong resistance. Besides, there is a need for additional sources of genetic resistance and its characterization, which can be obtained through germplasm screening.

The new approach of crop improvement may be exploited to investigate if there are any microbial species that may suppress the growth of *Alternaria solani* or *A. alternate*. Alternatively, there may be an enhanced level of resistance in tomato in the presence of certain microbes, which needs to be investigated. This is a completely new area of investigation. 

## Figures and Tables

**Table 1:** Genetic source of resistance to early blight in tomato identified in various parts of the world over time.

Source of Resistance	Resulting EB-Resistant Genotype	Type of Resistance	Reference
*Solanum lycopersicum*			--
Unknown source	C1943		[84]
68B134	71B2		[57]
C1943	NC63EB		[66]
C1943	NC870		[66]
C1943	NC EBR-2	Quantitative	[66]
71B2	NCEBR-5, NCEBR-6	Quantitative	[67]
Unknown accessions	HRC90.145, HRC90.158, HRC90.159		[73]
NC-EBR-1	NC EBR-4, IHR1816	Quantitative	[68,77]
NCEBR-1 and -2	NCEBR-3		[68]
NCEBR-3 and -4	Mountain Supreme		[68]
NCEBR-5 and -6	Plum Dandy		[67]
NCEBR-6	Mountain Magic		[70]
PI 406758	-		[89]
*Solanum habrochaites*			
PI127827	-		[72]
PI390514, PI390662	-		[89]
PI126445	NC EBR-1		[66]
PI1390662	87B187		[76]
B6013	H-7, H-22, H-25		[74]
Unknown accessions	HRC90.303, HRC91.279, HRC91.341		[73]
LA2100, LA2124, LA2204, PE36	-		[73]
PI126445	NC39E		[90]
PI390513	-		[89]
PI390516	-		[89]
PI390658	-		[89]
PI390660	-		[89]
LA2650	-		[73]
*Solanum peruvianum*			
PE33	-		[73]
LA1292	-		[73]
LA1365	-		[73]
LA1910	-		[73]
LA1983	-		[73]
PI270435	-		[73]
*Solanum pimpinellifolium*			
PI 365912, PI 390519	-		[89]
A 1921	P-1		[74]
L4394 (IHR1939)	-		[77]
Resistance to collar rot			
-	Devon Surprise		[71]
-	Red Cherry		[71]
-	Red Pear 414		[71]
-	Red Pear 415		[71]
-	Targinnie Red		[71]
-	Vetomold		[71]
-	Montgomery		[71]
-	Norduke		[71]
-	Marglobe		[71]
PI179532	-		[75]
PI127833	-		[75]

**Table 2:** Quantitative trait loci (QTLs) associated with early blight in tomato identified in various studies. RIL, recombinant inbred line.

No.	QTLs	Molecular Markers Associated	LOD Scores	*R*^2^-Value (%)	Parents	Type of Population	Population Size	References
1	chr1	TG559-TG208A	7	21.9	NC84173 × PI126445	BC_1_	145	[90]
	chr2	TG337-CT59	2.9	15.3	(*S. lycopersicum* × *S. habrochaites*)			
	chr5	XLRR.370-CT202	2.6	8.4				
	chr8	CD40-TG16	2.3	7.4				
	chr9	RLRR.130-CLRR.950	4.2	14.9				
	chr9	TG424-TG429	5.1	16.2				
	chr10	TG241-TG403	6.8	20.2				
	chr11	CT168-TG508	3.8	13.2				
	chr11	TG147-A41.3	2.3	7.4				
	chr12	CT100-TG68	3.1	10.3				
	chr12	AN23.390-TG180	4.1	12.9				
	chr1	TG559-TG208A	3.6	11.9	NC84173 × PI126445	BC_1_S_1_	145	[90]
	chr2	TG337-CT59	2.8	15.9	(*S. lycopersicum* × *S. habrochaites*)			
	chr3	TG411-TG214	2.9	9.1				
	chr5	TG441-CT242	2.6	7.9				
	chr5	XLRR.370-CT202	3.7	11.3				
	chr8	CD40-TG176	3	10.3				
	chr8	TG330-TG294	5.4	21.0				
	chr9	RLRR.130-CLRR.950	8.2	25.9				
	chr9	TG424-TG429	3.7	16.2				
	chr10	TG241-TG403	5.6	16.3				
	chr11	TG508-TG651	3.8	11.5				
	chr11	CT55-CD17	3	9.9				
	chr12	CT100-TG68	2.5	8.3				
2				*qR_S* (difference between resistant and susceptible allele frequency)	NC84173 × PI126445	BC_1_	820	[93]
	chr3	TG66, TG621		≤−0.22		(selective genotyping)		
	chr4	TG652, PK34-340		≤0.28				
	chr5	XLRR-370, RLRR-220, S23-300, PK12-340, CT202, TG318, CT172, CT118A, TG351, CT80A, TG185		≤0.32				
	chr6	AN23-410, CT825		≤0.23				
	chr8	TG46, TG330, TG36C, CT265, CT68, TG294		≤0.22				
	chr10	CT57, CEL79		0.21				
	chr11	TG497		0.19				
3		COFACTOR		RAUDPC (%)	Solentos × LA2157	F2 and F3	176	[25]
	chr1	P14M60-276P	4.1	6.8	(*S. lycopersicum* × *S. arcanum*)			
	chr2	P14M51-146E	9	16.2				
	chr5	P14M51-055P	6.2	10.5				
	chr6	P11M48-266E	6.3	10.8				
	chr7	P15M62-349P	8.3	15.2				
	chr9	P11M60-109P	8.7	15.5				
4	chr2, chr3, chr4, chr5, chr6, chr7, chr9, and chr12	-	-	combined effect = 44%	NCEBR1 × LA2093	F2, F3, F4	-	[18]
				Individual = 7.6% to13.4%	(*S. lycopersicum* × *S. pimpinellifolium*)			
5	chr1, chr2, chr3, chr4, chr5, chr6, and chr11	-	-	-	NCEBR1 × LA2093	F2	-	[18]
						(Selective genotyping)		
6	chr2	cLEC73K6b, cLEC73I19a, CT205	3.3	8.0	NCEBR1 × LA2093	RIL	172	[80]
	chr2	TG463, cTOFC19J9, CT103	2.9	5.5				
	chr5	cLEY-18H8, cTOA24J24, cTOC2J24, cTOC20J21	5.5	15.3				
	chr6	TG274, cLEN10H12, TG590, cLEN10H12	4.4	13.0				
	chr9	TG343, cLED4N20, TG348, cTOE10J18	4	11.5				

LOD = Likelihood of odd; QTL = Quantitative trait loci.
